# Joining the locals: Plant invaders shift leaf defenses to match native neighbors

**DOI:** 10.1002/ecy.70129

**Published:** 2025-06-08

**Authors:** Jason Fridley, Robert J. Griffin‐Nolan, Lamine Bensaddek, Guillaume Decocq, Kouki Hikosaka, Thomas Kichey, Julie LeVonne, Masako Mishio

**Affiliations:** ^1^ Department of Biology Syracuse University Syracuse New York USA; ^2^ Department of Biological Sciences Clemson University Clemson South Carolina USA; ^3^ Department of Biological Sciences California State University Chico California USA; ^4^ Unite Ecologie et Dynamique des Systemes Anthropises Universite de Picardie Jules Verne Amiens France; ^5^ Graduate School of Life Sciences Tohoku University Sendai Miyagi Prefecture Japan

**Keywords:** alkaloids, biological invasions, enemy release, evolution of increased competitive ability, glycosides, growth‐defense theory, plant traits, shifting defense theory

## Abstract

Local adaptation is common in invasive plants, but there is no consensus as to whether shifts in functional traits between invader “home” and “away” ranges contribute to their success in competition with native species. Theory based on enemy release suggests that invaders should reallocate limiting resources away from nutritive‐based defenses and toward high growth potential. However, empirical studies of home–away trait shifts are typically conducted on a single invader and fail to consider how environmental differences across regions may impact leaf trait syndromes. We measured nine defense‐related leaf traits for 27 invasive species across their home and away ranges in France, Japan, and the United States, and compared them to distributions of those same traits for co‐occurring native species in both their home and away ranges. Our study included a total of 21 woody species sampled under forest canopies, and 23 herbaceous species sampled in fields and roadsides. Traits included toxic leaf alkaloids and cyanogenic glycosides; structural attributes including cell wall mass and fiber content; carbon (C), nitrogen (N), C:N, and total protein content; and specific leaf area. We found significant overall shifts in both qualitative (alkaloids) and quantitative (fiber, cell wall, N content) defense traits, particularly in woody species that are hypothesized to be more apparent to herbivores. However, the direction of trait shifts was not consistent across regions. Rather, for seven of nine traits, trait means of invaders shifted toward the means of native species in the recipient communities, likely reflecting environmental differences among regions rather than a shift in allocation from defense to growth. We suggest this “join the locals” pattern, whereby trait shifts in invaders match regional differences in native trait syndromes due to environmental variation, is a reasonable null model for studies of adaptive evolution in invasive species. Although the “join the locals” pattern is not mutually exclusive with shifts in functional traits caused by enemy release, our study involving multiple species and habitats suggests environmental gradients override trait shifts driven by varying herbivore communities.

## INTRODUCTION

Herbivory is a primary driver of plant adaptive evolution (Züst & Agrawal, 2017). Plants exposed to novel herbivores may face local extinction (Brockerhoff & Liebhold, 2017), while those that escape co‐evolved enemies through human‐assisted introductions may outcompete native species in their introduced (“away”) range (Elton, 1958; Hawkes, 2007; Wolfe, 2002). Although such “enemy release” is not a contributor to all plant invasions (Liu & Stiling, 2006; Schultheis et al., 2015), herbivore manipulation experiments have shown beyond a doubt that enemy release contributes to invader advantage in some cases (DeWalt et al., 2004; Huang et al., 2012). What remains controversial is whether release from enemies furthers the adaptive evolution of introduced species in their away range (Felker‐Quinn et al., 2013; Müller‐Schärer et al., 2004). In this study, we investigate whether shifts in leaf defensive traits of invasive species are driven by release from natural enemies or reflect adaptation to the environmental conditions of the recipient region.

The evolution of increased competitive ability hypothesis (EICAH) is based on the reallocation of limiting resources such as nitrogen (N) away from defensive compounds and toward photosynthesis when specialist enemies are absent, contributing to enhanced carbon gain in invaders (Blossey & Notzold, 1995). Conversely, the shifting defense hypothesis (SDH) predicts that invaders in their away range increase defenses against generalist herbivores by upregulating toxic compounds (Zhang et al., 2018). Empirical studies remain equivocal on which traits, if any, commonly shift between invader home versus away ranges (Felker‐Quinn et al., 2013; Zhang et al., 2018). In part, this is because invader home–away studies are logistically difficult, focusing on single species and habitat types. There is also the question of which traits to measure, given that plant defensive strategies are multidimensional and include the synthesis of toxic defense compounds, the nutritional content of plant tissues, and their digestibility (Agrawal & Fishbein, 2006).

Certain plant defense traits may be more prone to away‐range evolution than others. Some secondary metabolites that are toxic to consumers, such as alkaloids and cyanogenic glycosides, are enriched in N and thus may constrain protein‐demanding functions such as photosynthesis if expressed in the absence of herbivores (Gleadow & Møller, 2014). These compounds are considered “qualitative” defenses because even low amounts offer protection from generalist herbivores that have not co‐evolved a means of reducing their toxicity (Müller‐Schärer et al., 2004). However, some have expressed doubt whether the low concentrations required to deter herbivores for most types of alkaloids (Cheng et al., 2017; Levin & York, 1978) and cyanogenic glycosides (Ballhorn et al., 2005; Patton et al., 1997) are sufficient to compete with other N‐limiting leaf functions (Gleadow & Møller, 2014; Koricheva, 2002). Moreover, introduced plants are more likely to be released from specialist rather than generalist enemies (Joshi & Vrieling, 2005; Müller‐Schärer et al., 2004), and the SDH suggests invaders should increase levels of defense against generalist herbivores in their away range by upregulating toxic defense compounds (Zhang et al., 2018). Although meta‐analyses of herbivore damage studies generally support the SDH (Doorduin & Vrieling, 2011; Zhang et al., 2018), empirical studies that quantify defensive metabolites in the invader home and away ranges are rare. Shifts in qualitative defenses have been documented in a few single taxon studies (e.g., Brandenburger et al., 2020; Griffin‐Nolan et al., 2024; Joshi & Vrieling, 2005), but their general applicability to invader advantage remains unclear.

In contrast to qualitative defenses, quantitative defense traits are those that deter herbivory through low tissue nutrient content or digestibility, and thus apply to both generalist and specialist herbivores (Müller‐Schärer et al., 2004; Oduor, 2022). Quantitative defense traits involve aspects of herbivore nutrition, such as protein or N content, as well as compounds that interfere with digestion, including cell wall and fiber content (lignin, cellulose, hemicellulose) and polyphenolic compounds such as tannins (Coley, 1988; Müller‐Schärer et al., 2004). Most quantitative defensive traits are negatively associated with photosynthetic function and potential growth rate, either directly (e.g., from low Rubisco content) or indirectly through their negative associations with specific leaf area (SLA; Müller‐Schärer et al., 2004). Many researchers have argued that reduced quantitative defenses in the away range of invaders, triggered by a loss in key specialist consumers, may explain why invaders often have greater Rubisco content, carboxylation rates, and photosynthetic N‐use efficiency (PNUE) than co‐occurring native species (Feng & Fu, 2008; Fridley et al., 2022; Ordonez & Olff, 2013). For example, Feng et al. (2009) showed that a shift toward lower cell wall mass in the away range of *Ageratina adenophora* was associated with greater N allocation to photosynthesis, and Huang et al. (2010) associated an increase in growth potential in the away range of *Triadica sebifera* with an increased protein‐to‐carbohydrate ratio and lower specialist herbivore loads. Although quantitative traits are generally easier to measure than concentrations of qualitative toxic compounds, studies of adaptive shifts in quantitative defense traits of invaders remain difficult to generalize because they focus on single species, and there remains a paucity of studies that consider both quantitative and qualitative defensive traits.

**FIGURE 1:**
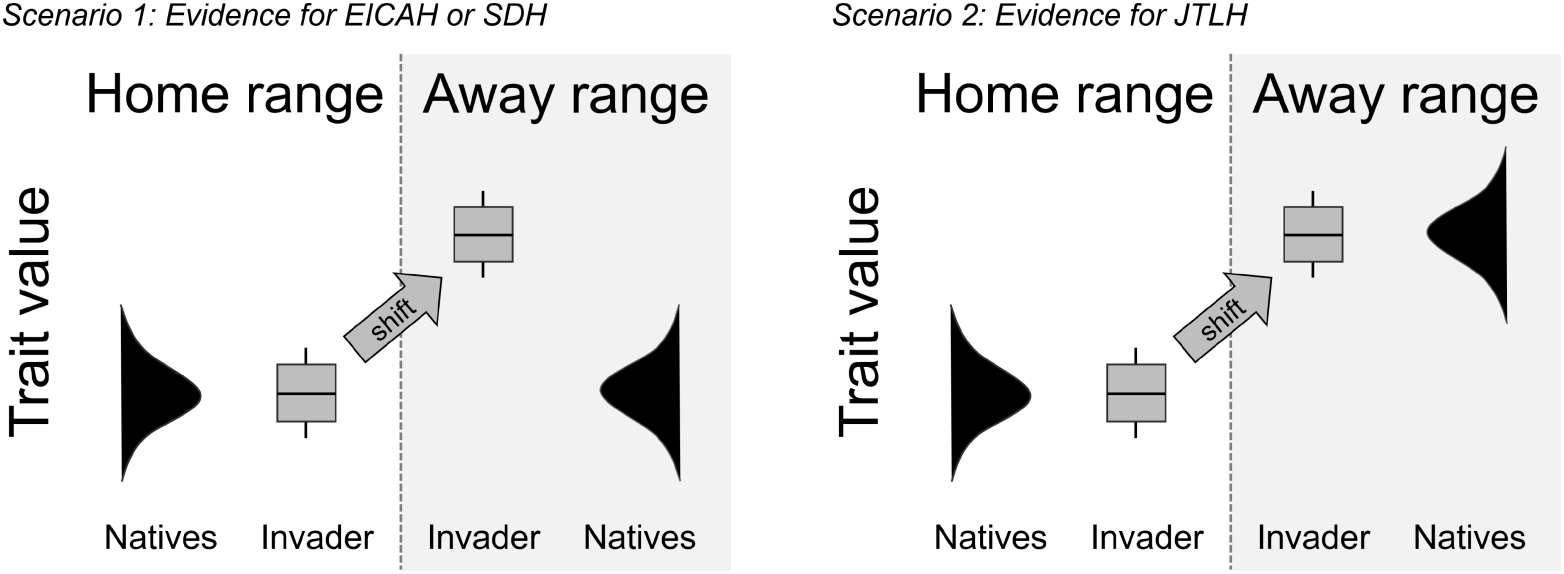
Conceptual diagram illustrating the “join the locals” hypothesis. We draw a distinction between shifts in leaf traits in invasive species driven by a loss of herbivores, versus those driven by environmental differences in the home and away ranges. If environmental properties that influence resource economics are similar in the home and away ranges, then a shift in trait distribution related to loss of herbivores (e.g., an increase in protein content) may support evolution of increased competitive ability hypothesis (EICAH) or shifting defense hypothesis (SDH). (Scenario 1: The trait values of the home and away communities are similar.) Alternatively, if traits in the away‐range native community are different from those of the home‐range native community of the invader, an associated home‐away shift in the same direction would support an environmental explanation rather than one based on enemy release (Scenario 2: “join the locals hypothesis,” JLTH).

One underexplored explanation of invader adaptive evolution is the role of local environmental and community context. Because plant defensive strategies are driven in part by the environment (Coley et al., 1985; Hahn et al., 2019), an alternative explanation of home–away shifts in defense‐related traits is that species are adapting to the local environment of their away range rather than altered herbivore loads. For example, low soil fertility is often associated with reduced concentrations of qualitative defenses, including N‐rich secondary metabolites like alkaloids and cyanogenic glycosides (Gleadow & Møller, 2014), as predicted by growth‐defense trade‐off theory (Züst & Agrawal, 2017). Similarly, leaf N and protein content should covary with other leaf economic traits that are sensitive to site fertility, light levels, climate, and other factors (Moreira et al., 2018; Onoda et al., 2011), which may in turn alter the expression of toxic compounds. Because the environments of home and away populations of invaders vary in numerous ways, it is difficult to disentangle environmental and biotic drivers of defense‐related shifts in studies of single species (Moles et al., 2013). One solution is to take a community approach and compare the expression of quantitative and qualitative defense traits in invaders to those of co‐occurring native species in both home and away populations. For example, for shifts in defensive traits in the away range of an invader to conclusively support EICAH or SDH, they should be greater than the mean difference of those traits of native communities in the home and away regions (Scenario 1 in Figure 1). In this case, one could infer that the invader is altering its phenotype because of altered consumption levels rather than environmental context. On the other hand, if native species in the away range already express different levels of a defensive trait based on regional environmental differences, one might expect the invader to adapt its defensive strategy to the away‐range environment (Scenario 2 in Figure 1). We call this pattern “joining the locals” hypothesis (JTLH) and suggest it can be statistically distinguished from EICAH and SDH by comparing traits across four populations: (1) other native species in the “home” community of the invader, (2) home populations of the invader, (3) populations of the invader in its away range, and (4) co‐occurring native species in the invader's away range. To our knowledge, no study has yet examined the context of invader functional traits (defensive or otherwise) across each of these populations.

**FIGURE 2:**
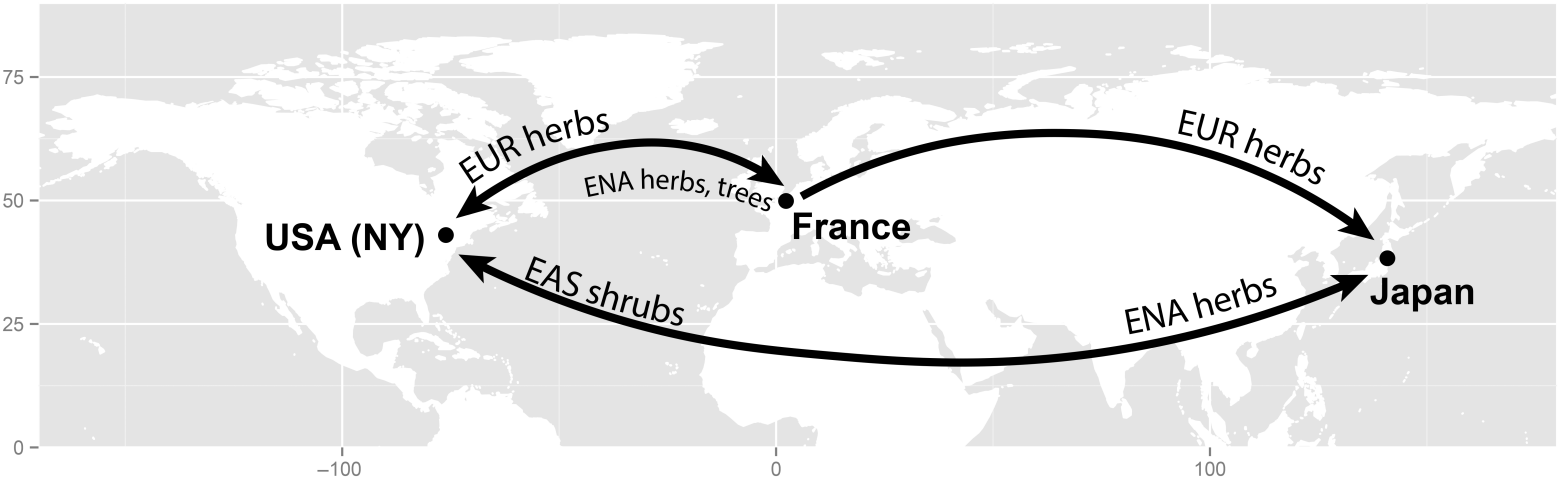
Focal regions and invasive species groups. We measured nine defense‐related traits for woody and herbaceous species in and around Central New York (NY) State, USA; Amiens, France; and Sendai, Japan. Invasive species were measured in their home and away ranges, including grasses and short‐lived forbs native to Europe (EUR herbs, 7 species); forest shrubs and lianas native to East Asia (EAS shrubs, 7 species); herbaceous meadow species native to Eastern North America (ENA herbs, 5 species); and woody trees native to ENA (ENA trees, 5 species). We measured an additional 17 native species across the three regions that co‐occur with the invaders in either their home or away ranges (Table 1).

Here, we demonstrate how defense‐related traits of woody and herbaceous invasive plants (Table 1) shift between their home and away ranges, and test whether trait shifts, when they occur, support the EICAH or the SDH. Specifically, we test whether quantitative traits reflecting nutrient content and digestibility—including leaf N, C, CN, total protein, fiber, and cell wall content (Table 2)—are more likely to decline in the away range of invaders than qualitative defensive traits, including alkaloid and cyanogenic glycoside content, which should increase according to the SDH. Reductions in either category of defensive traits in the away range could support the EICAH. As an alternative hypothesis of away‐range trait evolution, we also measured these same traits in native co‐occurring species in each region to test whether trait shifts instead support a “join the locals” model, where invaders shift trait values in the direction of the mean of native (recipient) communities. The generality of our study is buttressed by focusing on two habitat types and growth forms (woody species in forests, and herbaceous species in old fields, roadsides, and meadows) across three separate temperate regions of the Northern Hemisphere, including 27 invasive species from one or more regions in France, Japan, and the United States, and 44 species total (Figure 2, Table 1). We included invaders of different habitats and growth forms for two reasons. First, apparency theory (Feeny, 1976; Strauss et al., 2015) suggests constraints on leaf function from herbivory are generally greater in woody species than that of herbaceous species, due to the overall larger stature and lifespan of woody species that facilitate more effective foraging by herbivores (Haukioja & Koricheva, 2000; Smilanich et al., 2016). Second, growth‐defense theory (Coley et al., 1985) suggests slower‐growing plants in shaded understory environments would invest more in defenses than species of high‐light environments, where higher photosynthetic rates may favor herbivory tolerance rather than defensive allocation (DeWalt et al., 2004). Based on these considerations, we expected that home–away shifts in defensive traits, particularly quantitative defenses, would be greater in woody invaders in forest habitats.

**TABLE 1:** Focal species, including taxonomic family, number of populations sampled in each region, and where the species is native and invasive outside of its native range.

Species	Family	Type	Native to	Invasive in	No. populations sampled		
					France	Japan	USA
*Daucus carota*	Apiaceae	Herbaceous	France	…	5	0	0
*Eupatorium cannabinum*	Asteraceae	Herbaceous	France	…	5	0	0
*Senecio jacobaea*	Asteraceae	Herbaceous	France	…	5	0	0
*Senecio vulgaris*	Asteraceae	Herbaceous	France	…	5	0	0
*Humulus lupulus*	Cannabinaceae	Woody	France	…	5	0	0
*Laburnum anagyroides*	Fabaceae	Woody	France	…	6	0	0
*Quercus robur*	Fagaceae	Woody	France	…	5	0	0
*Acer pseudoplatanus*	Sapindaceae	Woody	France	…	6	0	0
*Chenopodium album* ^a^	Amaranthaceae	Herbaceous	France	Japan/USA	6	4	0
*Leucanthemum vulgare*.^a^	Asteraceae	Herbaceous	France	Japan/USA	5	10	5
*Plantago lanceolata* ^a^	Plantaginaceae	Herbaceous	France	Japan/USA	6	5	5
*Agrostis gigantea* ^a^	Poaceae	Herbaceous	France	Japan/USA	5	5	0
*Agrostis stolonifera* ^a^	Poaceae	Herbaceous	France	Japan/USA	5	5	0
*Artemisia vulgaris* ^a^	Asteraceae	Herbaceous	France	USA	5	0	5
*Anthoxanthum odoratum* ^a^	Poaceae	Herbaceous	France	USA	5	5	0
*Dactylis glomerata* ^a^	Poaceae	Herbaceous	France	USA	5	0	0
*Rhamnus cathartica* ^a^	Rhamnaceae	Woody	France	USA	5	0	6
*Prunus avium* ^a^	Rosaceae	Woody	France	USA	6	0	4
*Solidago virgaurea*	Asteraceae	Herbaceous	France/Japan	…	3	5	0
*Cirsium japonicum*	Asteraceae	Herbaceous	Japan	…	0	4	0
*Eupatorium glehnii*	Asteraceae	Herbaceous	Japan	…	0	5	0
*Youngia japonica*	Asteraceae	Herbaceous	Japan	…	0	4	0
*Plantago asiatica*	Plantaginaceae	Herbaceous	Japan	…	0	5	0
*Viburnum dilatatum* ^a^	Adoxaceae	Woody	Japan	USA	0	5	5
*Berberis thunbergii* ^a^	Berberidaceae	Woody	Japan	USA	0	8	5
*Lonicera japonica* ^a^	Caprifoliaceae	Woody	Japan	USA	0	4	5
*Lonicera morrowii* ^a^	Caprifoliaceae	Woody	Japan	USA	0	3	5
*Celastrus orbiculatus* ^a^	Celastraceae	Woody	Japan	USA	0	4	5
*Euonymus alatus* ^a^	Celastraceae	Woody	Japan	USA	0	5	4
*Rosa multiflora* ^a^	Rosaceae	Woody	Japan	USA	0	4	5
*Bidens tripartita*	Asteraceae	Herbaceous	USA	…	3	0	0
*Viburnum acerifolium*	Adoxaceae	Woody	USA	…	0	0	5
*Lonicera canadensis*	Caprifoliaceae	Woody	USA	…	0	0	4
*Lindera benzoin*	Lauraceae	Woody	USA	…	0	0	5
*Robinia pseudoacacia* ^a^	Fabaceae	Woody	USA	France	6	0	10
*Quercus rubra* ^a^	Fagaceae	Woody	USA	France	5	0	5
*Prunus serotina* ^a^	Rosaceae	Woody	USA	France	5	0	5
*Acer negundo* ^a^	Sapindaceae	Woody	USA	France	5	0	10
*Parthenocissus quinquefolia* ^a^	Vitaceae	Woody	USA	France	7	0	5
*Ambrosia artemisiifolia* ^a^	Asteraceae	Herbaceous	USA	France/Japan	5	5	5
*Bidens frondosa* ^a^	Asteraceae	Herbaceous	USA	France/Japan	5	5	3
*Conyza canadensis* ^a^	Asteraceae	Herbaceous	USA	France/Japan	6	5	6
*Erigeron annuus* ^a^	Asteraceae	Herbaceous	USA	France/Japan	5	5	4
*Solidago gigantea* ^a^	Asteraceae	Herbaceous	USA	France/Japan	5	4	6

^a^ These species are invasive in at least one region and were sampled in their home and away ranges.

**TABLE 2:** List of measured traits.

Trait (units)^a^	Region			df	*F*	*p*
	Japan	France	USA			
Cyan (eq. mg KCN g^−1^)	15.66 ± 0.06ab	15.76 ± 0.05a	12.61 ± 0.06b	2, 369	4.32	0.0139
Alkaloids (eq. mg atropine g^−1^)	0.34 ± 0.08a	0.30 ± 0.08a	0.57 ± 0.10b	2, 383	13.70	<0.001
Total fiber (%)	41.40 ± 0.12a	40.90 ± 0.01a	39.02 ± 0.02a	2, 320	1.24	0.291
Cell wall mass (mg cm^−2^)	0.85 ± 0.06a	1.35 ± 0.09b	0.75 ± 0.06a	2, 365	9.10	<0.001
Total protein (mg cm^−2^)	0.60 ± 0.04a	0.87 ± 0.06b	0.79 ± 0.03b	2, 384	8.57	<0.001
CN	23.60 ± 0.02a	18.68 ± 0.03b	21.06 ± 0.03c	2, 361	22.86	<0.001
C_mass_ (%)	44.24 ± 0.01a	43.70 ± 0.01a	45.50 ± 0.01b	2, 361	7.99	<0.001
N_mass_ (%)	2.00 ± 0.25a	2.61 ± 0.28b	2.34 ± 0.28b	2, 366	20.1	<0.001
SLA (cm^2^ g^−1^)	268 ± 4.1a	267 ± 2.7a	330 ± 3.3b	2, 340	21.58	<0.001

*Note*: Values are mean (±1 SE) within regions (including native and invasive species). *F* statistics are from one‐way ANOVAs with region as the predictor (including numerator and denominator df and *p* value); letters indicate significant pairwise differences (*p* < 0.05) according to Tukey's honestly significant difference tests (Appendix S1: Table S4).

Abbreviation: SLA, specific leaf area.

^a^ N_mass_ and C_mass_ are leaf N and C concentrations, respectively, on a mass basis; CN is the ratio of leaf C to N; total protein is the sum of water and sodium dodecyl sulfate (SDS)‐soluble protein on a fresh area basis; cell wall mass is on a fresh area basis; total fiber (in percentage) is the sum of dry leaf mass proportions of hemicellulose, cellulose, and lignin; alkaloids is total alkaloid concentration; cyan is total cyanogenic glycoside concentration (*Prunus serotina* excluded).

## METHODS

### Target species

Our study included three regions of the Northern Hemisphere that are climatically similar but, before ca. 1500 C.P.E, had been floristically isolated since the Miocene (Fridley, 2013). The Somme department of the Hauts‐de‐France region of northern France (49.9° N, 2.3° E; Figure 2) includes the home ranges of European herbaceous species that were spread into disturbed habitats in the temperate zone worldwide (Fridley, 2008), including grasses and short‐lived forbs often found on roadsides and other sites of chronic disturbance. We sampled eight of these species (Table 1) in both their home range (around Amiens) and at least one of their away ranges in the United States and Japan. We also sampled 9 co‐occurring native species (4 woody and 5 herbaceous) in this region that are not invasive in the other two regions. The Miyagi and Fukushima prefectures of Japan on the island of Honshu (38.3° N, 140.9° E; Figure 2) are within the home range of many shrubs and lianas that have become invasive in forests of the Northeastern United States (Fridley, 2008). Although few global herbaceous invaders are native to this region, it is the recipient of herbaceous invaders from both Europe and the United States, the latter being more typically longer‐lived, clonal perennials than those from Europe (Fridley, 2013). We sampled seven woody species native to Japan that are invasive in the United States (Figure 2), and 10 herbaceous species in this region introduced from France or the United States (Table 1). We also sampled five co‐occurring native herbaceous species in this region that are not invasive in the other two regions. Finally, the Syracuse region of central New York State (USA, 43.0° N, 76.1° W; Figure 2) includes forested areas heavily invaded by shrubs and lianas from Japan, and old fields and roadsides dominated by European herbs and grasses (Fridley, 2013). It is also the home range of herbaceous meadow species invasive in France and Japan, as well as several tree species invasive in Europe. We included in this study five woody and five herbaceous species native to the United States but invasive in France and/or Japan (Table 1). We also sampled 4 co‐occurring native species (3 woody, 1 herbaceous) in this region that are not invasive in the other two regions.

Overall, our study included 44 species, including a total of 414 populations across three continents. Our target species included 21 woody species sampled under a forest canopy in full or partial shade and 23 herbaceous species sampled in full sunlight. Of the 44 species, 14 woody species and 13 herbaceous species were invasive in at least one of our focal regions, and we sampled these species in both their home and away ranges. Of the herbaceous species, seven were present and sampled in all three regions (Table 1), giving a total of 33 home–away contrasts. We also sampled 17 native species that co‐occur with one or more invaders in each region, allowing comparisons of traits between invaders and neighboring native species. Where possible, we chose native species of similar taxonomy and functional guild (e.g., association with N‐fixing bacteria). We did not attempt to match environmental variables across regions due to practical difficulties in finding populations of the same species across native and invasive ranges and the multidimensional nature of environmental data. Our goal was to sample at least five separate populations of each species within each region, although travel constraints during 2019–2021 limited our replication to 3 or 4 populations for some species and regions (Table 1).

### Field sampling

From 2019 to 2021, we sampled mature, healthy leaves from natural populations in each region, from June to the first week of September, depending on species' phenology. We avoided sampling leaves with obvious herbivore or pathogen damage. For most species, we sampled 4–5 leaves from each population from 1 or 2 individuals; we doubled this amount for smaller‐leaved forbs and grasses. We measured SLA (in square centimeters per gram) as fresh leaf area (scanned at 300 dpi and analyzed using ImageJ software, https://imagej.nih.gov/ij/) divided by oven‐dried mass, averaged over the sampled leaves. These leaves were ground into a fine powder for shipment to the Cornell Stable Isotope Laboratory (Cornell University, Ithaca, NY, USA) to measure mass‐based leaf carbon and nitrogen content. For each population, we measured the pH of A horizon mineral soils in the laboratory with a glass electrode in soil‐water suspension (Miller & Kissel, 2010). For each population in forested sites, we estimated light availability at the height of the highest surveyed leaf using hemispherical photography following the method of Martinez et al. (2019). Values are expressed as a Gap Light Index (GLI), from 0% to 100% light (Frazer et al., 1999).

### Leaf protein and cell wall mass

We determined total leaf protein content, cell wall mass, and cell wall N content following Hikosaka and Shigeno (2009). Leaf disks (15–20) from an additional 2–3 leaves from each population were wrapped in foil, frozen in liquid N, and stored at −80°C on the day of sampling. Five leaf disks (2.5 cm^2^) per sample were homogenized in a Bicine buffer (0.1 M Bicine pH 8.0, 10 mM dithiothreitol (DTT), 1% polyvinylpyrrolidone) at low temperature (<4°C) using a temperature‐controlled bead mill. The homogenate was centrifuged at 15,000 rpm for 30 min, and the supernatant containing water‐soluble proteins was removed. Following treatment with trichloroacetic acid (TCA), which removes DTT, the supernatant was assumed to contain only water‐soluble proteins. The remaining pellet was homogenized in an sodium dodecyl sulfate (SDS) buffer (0.1 M Bicine pH 8.0, 2% SDS) and centrifuged at 15,000 rpm (20°C) for 30 min. The supernatant (regarded as SDS‐soluble proteins) was collected, and this process was repeated a total of three times. We determined the protein concentration of both the water‐soluble and SDS‐soluble fractions via spectrophotometry using the Pierce BCA protein assay kit (Thermo‐Fischer Scientific) with bovine serum albumin used to make calibration curves. The remaining pellet from the final centrifugation was treated with amyloglucosidase solution to remove starch and phenol:acetic acid:water solution (PAW, 2:1:1 volume ratio) to remove nitrogenous compounds other than cell wall proteins. The final pellet was washed with water and then 100% ethanol before being oven‐dried (1 week at 70°C) and weighed to measure cell wall mass. This pellet was then measured for N content via an elemental autoanalyzer as above.

### Fiber content

Dried leaves from all sampled individuals were analyzed for total fiber content in Syracuse, NY, following the protocol of Hinman et al. (2019). Dried and ground leaf material (0.5 g per individual) was ground to pass through a 40‐mm mesh screen (Thomas Scientific Mini Wiley Mill, Swedesboro, NJ, USA), placed in a heat‐sealable ANKOM filter bag (ANKOM Technology, Macedon, NY, USA), and subjected to sequential digestions. These included (1) a 75‐min soak in a warm neutral detergent solution (3% sodium lauryl sulfate, 2% EDTA, 1% triethylene glycol) to remove soluble proteins and non‐structural carbohydrates; (2) a 60‐min soak in acid detergent wash (3% sulfuric acid) to remove hemicellulose and cell membrane‐bound proteins; and (3) a 3‐h soak in 72% sulfuric acid to remove cellulose from the samples, leaving behind lignin and minerals. Following each digestion, bags were oven‐dried for at least 12 h at 105°C and weighed to determine the percent loss of leaf dry mass. Mineral mass was subtracted from each final sample by mass loss through combustion at 500°C for 5.5 h. We added fractions of hemicellulose, cellulose, and lignin to estimate the total fiber content (in percentage) of each sample.

### Alkaloids and cyanogenic glycosides

Secondary metabolites were analyzed in Amiens, France, using a separate sample of 3 g of freeze‐dried leaf mass from each population. Each sample was assayed twice for each metabolite, and standard solutions were made fresh daily. To determine the total alkaloid content, 250 mg of ground leaf material was mixed with 10 mL of 0.05 M H_2_SO_4_ in a screw‐capped tube for 90 min at 80°C in an ultrasonic bath (Wagner & Bladt, 1996). This extract was alkalized to a pH of 9 with 28% NH_4_OH, then stirred and absorbed on 12 mL of kieselguhr in a 20‐mL column and left overnight. Five hundred microliters of 28% NH_4_OH was added to the column, and total alkaloids were eluted with 2 × 5 mL CHCl_3_. Combined fractions were transferred to an Erlenmeyer flask containing 5 mL of bromocresol green (BG) solution (John et al., 2014). Samples were stirred for 5 min before the addition of 5 mL of a phosphate buffer (pH 4.7), then stirred for another 20 min to achieve complexation. Non‐miscible phases were decanted in a separation funnel for 105 min. The organic (lower) phase containing the alkaloid‐BG complex was transferred to a glass tube and placed in the dark overnight. The absorbance at 470 nm was compared to an atropine sulfate salt monohydrate standard calibration curve with CHCl_3_ as a blank. Values of alkaloid concentration are in units of milligrams of atropine equivalents per gram leaf.

Cyanogenic glycoside concentration was measured on the same leaf source material as alkaloids, using combined autolysis and ezymatic hydrolysis (Brinker & Seigler, 1989). We extracted 100 mg of leaf powder in a 2‐mL tube containing 1.5 mL of citrate buffer (pH 5.5) with 10 μL of 40 u mL^−1^ β‐glucosidase solution (Sigma–Aldrich Merck chemicals), maintained at 37°C for 8 h. Two hundred microliters of 2 M NaOH was added to stop the hydrolysis and trap the cyanide ion. Extracts were centrifuged (3900 g) for 90 min, and 50 μL of the supernatant was added to 1 mL citrate buffer (pH 5.5) and 60 μL of Chloramine T solution in a 2‐mL tube to complex the cyanide ions with chlorine, forming CNCl. After 5 min, we added 225 μL of isonicotinic‐barbituric acid solution following Bradbury et al. (1994). After 40 min at 35°C, the reaction was complete, and the absorbance of the resulting polymethine dye was determined at 600 nm (Maranna et al., 2018). The samples were compared to a KCN calibration curve, resulting in values of cyanogenic glycoside concentration in units of millgrams KCN equivalents per gram leaf.

### Statistical analysis

All statistical tests were performed in R 4.3.0 (R Development Core Team, 2023). All traits were log‐transformed in all analyses to correct for strong positive skew. Log transformation of cell wall mass increased rather than decreased skew, so it remained untransformed.

#### Univariate

We used hierarchical Bayesian (HB) models to estimate effect sizes of the change in trait values between home and away ranges. This approach allowed us to estimate changes in multiple traits simultaneously without concerns of multiple testing, and it allowed us to use all species and populations regardless of the presence of missing values. Furthermore, HB modeling in JAGS (Plummer, 2003) estimates missing values in Y as posterior distributions by default, allowing us to use all observations (observed and estimated) in multivariate analysis (see below). We incorporated within‐species autocorrelation as a random intercept, and we allowed the home–away effect to vary according to the native region of each species (random slope) to account for region‐specific effects. We excluded one woody species, *Prunus serotina*, from the cyanogenic glycoside model because it had values two orders of magnitude higher (>300 eq. mg KCN g^−1^) than all other species in both its home and away ranges. Models took the form:

$$Y_{i,j}\sim N\left(\mu_{i,j},{\sigma_{\mathrm{Yj}}}^{2}\right)$$

$$\mu_{i,j}=\alpha_{j,s}+\beta_{j,r}{\text{Home}}_{i}$$

$$\alpha_{j,s}\sim N\left(0,{\sigma_{\mathrm{Sj}}}^{2}\right);\beta_{j,r}\sim N\left(0,{\sigma_{\mathrm{Rj}}}^{2}\right)$$

where *Y*_*i*,*j*_ is the value of trait *j* for individual *i*; *s* and *r* are the species and native region of individual *i*; and Home takes values of 0 or 1 for away and home populations. Effects of species on each trait were modeled according to a normal distribution of mean 0 and variance σ_*Sj*_^2^; and effects of native region on the home–away effect for each trait were modeled normally of mean 0 and variance σ_*Rj*_^2^. Non‐informative priors for normal variance were modeled according to uniform distributions (0–100). Before modeling, each trait (*Y*) was *Z*‐standardized to unit variance and mean 0 to facilitate effect size comparisons across different units. Models were fit by JAGS from the R2jags package in R (Su & Yajima, 2021). We confirmed model convergence with the Gelman and Rubin (1992) test statistic after three chains of 5000 iterations each and a 500‐iteration burn‐in. We estimated *R* ^2^‐like goodness of fit of each trait model following Gelman et al. (2019), including separate *R* ^2^ estimates for models with (“conditional”) and without (“marginal”) species‐level random effects that often dominated explained variance. To avoid the need for interpreting interaction terms, we modeled woody and herbaceous species separately, and compared trait differences between woody and herbaceous species with Student's *t* tests.

To test the “join the locals” hypothesis, for each trait we compared invader home–away differences to the mean difference of native species of the same regions. For calculating the native species means, we only included species of the same growth form (woody vs. herbaceous), and we did not include the target invader species' home trait value to avoid circularity. For example, the SLA of *Lonicera japonica* in its native range in Japan had a mean value of 282.4 cm^2^ g^−1^, which increased to a mean of 338.1 in the United States. However, native woody species in Japan (excluding *L. japonica*) had a mean SLA of 302.4, whereas those of the United States had a mean SLA 354.6. Thus, *L. japonica*'s trait shift is consistent with a “join the locals” hypothesis; if this is generally true for other species and regions, the overall relationship of invader and native region trait differences should be positive. We plotted this relationship for each trait, which included 33 possible invader‐region samples along with the associated mean native differences. Given the samples are not independent because native contrasts often share the same species, we used a bootstrap technique to test whether these relationships were significantly positive, whereby we randomly sampled with replacement each *X* value (regional difference) and *Y* value (invader shift) 1000 times, and calculated the bootstrapped *p* value as the proportion of times the observed slope was greater than the slope calculated from shuffled data (Fridley et al., 2004).

We used ANOVA to compare environmental variables (soil pH, GLI, mean annual temperature [MAT] and precipitation [MAP]) across regions. Site‐specific values of MAT and MAP were derived from 30‐year normals (1970–2000) of each site using 2.5‐min gridded climate data (Fick & Hijmans, 2017).

#### Multivariate

We analyzed woody and herbaceous species trait datasets in separate principal components analyses (PCA) using the “princomp” function in base R on Z‐scaled variables. We replaced missing trait observations in the raw dataset with the mean of their posterior distributions from their respective HB models (above). We tested whether the first two principal components (PC1, PC2) differed significantly in home versus away populations using a mixed model that included fixed effects of home–away, native region, and their interaction, plus a random intercept to account for within‐species correlation. This model is parallel to the structure of the univariate HB models (above), except that we treated the interaction of native‐range and home–away effects as fixed effects because random slope models would not converge. Mixed models were fit using the “lmer” function in the R package lme4 (Bates et al., 2015).

## RESULTS

### Environmental differences across regions

Environmental variables varied significantly across regions (Table 3). Soil pH was narrowly distributed within France with a relatively high mean of 7.3, had intermediate values in the United States (6.2), and was relatively acidic in Japan (5.1). GLI was lowest in the United States (mean 10%) and highest in Japan (25%). The climate of sites in Japan was slightly warmer (11.0°C MAT) than that of France (10.0°C) and much warmer than that of the United States (8.2°C). However, US sites were wetter (1100 mm MAP) than those of France (680 mm), and both were drier than those of Japan (1300 mm).

**TABLE 3:** Mean ± SE soil pH, Gap Light Index (GLI), mean annual temperature (MAT), and mean annual precipitation (MAP) across regions.

Variable	Region			df	*F*	*p*
	Japan	France	USA			
Soil pH	5.1 ± 0.15a	7.3 ± 0.07c	6.2 ± 0.20b	2, 107	70.1	<0.001
GLI (%)	25 ± 4.8a	19 ± 2.0ab	10 ± 1.8b	2, 30	6.0	0.0063
MAT (°C)	11 ± 0.36a	10 ± 0.04a	8.2 ± 0.18b	2, 117	56.2	<0.001
MAP (mm)	1300 ± 23c	680 ± 4.2a	1100 ± 9.0b	2, 117	853.3	<0.001

*Note*: *F* statistics are from one‐way ANOVAs with region as the predictor (including numerator and denominator df and *p* value); letters indicate significant pairwise differences (*p* < 0.05) according to Tukey's honestly significant difference tests (Appendix S1: Table S5). GLI was only measured for forested plots.

### Differences in defense traits across regions and growth forms

We found significant regional differences in all traits measured except for leaf total fiber content (Table 2). Cyanogenic glycosides (excluding *P. serotina*) were higher in France than in the United States, while alkaloids were higher in the United States than in both France and Japan. Cell wall mass was higher in France than in the other two regions, while leaf C and SLA were highest in US populations. Total protein and N content were lower in Japan than in the other two regions. CN ratios were different across regions, with those in Japan the highest and those in France the lowest. Although cyanogenic glycoside content was more than two orders of magnitude higher for *P. serotina* in both its home and away ranges (mean = 306 eq. mg KCN g^−1^), values were consistent across ranges (Student's *t* test, *t* = −0.546 on 7.964 df, *p* = 0.60).

**FIGURE 3:**
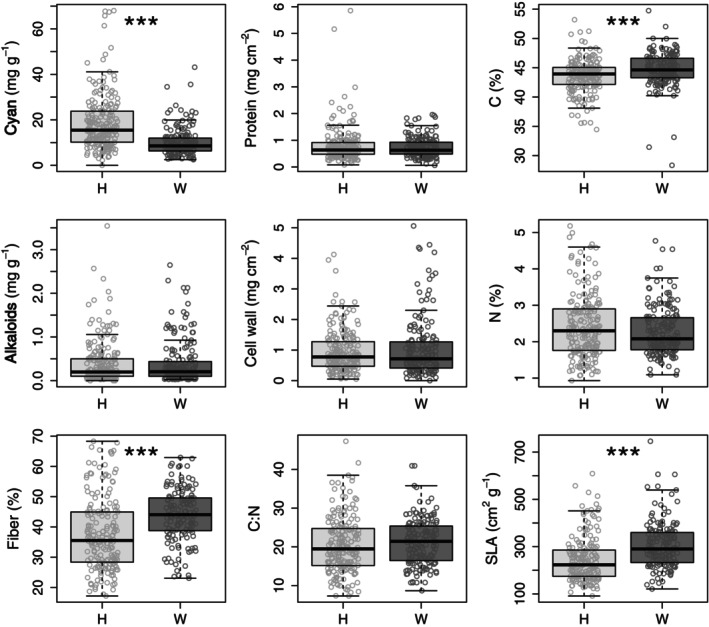
Distribution of nine leaf defensive traits for herbaceous (H) and woody (W) individuals across regions. Boxes show middle quartiles with median midline, whiskers show data range, and points are outliers. Asterisks indicate *p* values (***p < 0.001) of Student's *t* tests evaluating the null hypothesis that distributions are of equal means (Appendix S1: Table S1). Cyanogenic glycosides and alkaloids are considered qualitative defenses and labeled in bold, and the rest are quantitative. SLA, specific leaf area.

Overall, woody species had greater leaf C and fiber content than herbaceous species (Figure 3, Appendix S1: Table S1), although the effect size was greater for fiber content (43.9% vs. 37.7%) than leaf C (44.7% vs. 43.6%). We also found leaves of woody species to have significantly greater SLA (306 vs. 246 cm^2^ g^−1^) but lower cyanogenic glycosides (10.1 vs. 18.5 eq. mg KCN g^−1^) than those of herbaceous species (Figure 3, Appendix S1: Table S1).

### Shifts in defense traits between invader home and away ranges

**FIGURE 4:**
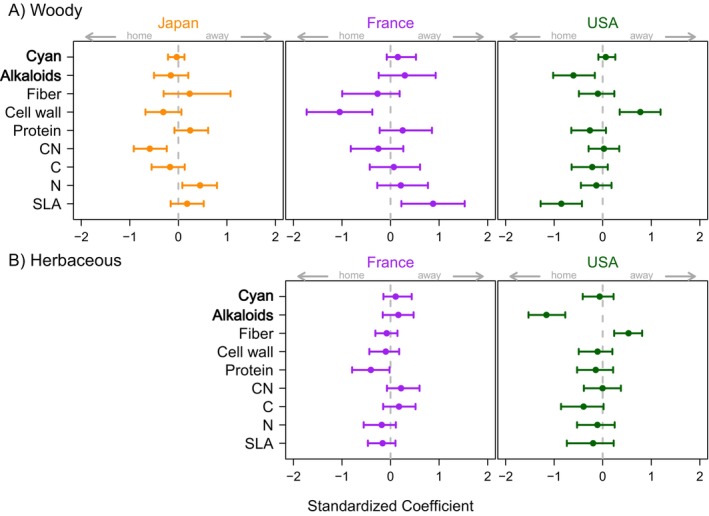
Evidence for shifts in leaf traits between home and away ranges, in separate analyses for (A) woody and (B) herbaceous species. Panels separate species by their home range. Midpoints represent the posterior mean and bars are 95% credible intervals of the difference between home and away populations; positive values indicate larger trait values for away populations. Traits (*Y* axis) are listed in Table 2. Coefficients were allowed to vary according to the region of origin of each species. In our study, there were no invasive herbs native to Japan. Marginal and conditional *R* ^2^ values of each trait are listed in Appendix S1: Table S2. Qualitative defenses are labeled in bold. SLA, specific leaf area.

Summaries of each trait for all invasive species across their home and away populations are reported in Appendix S1: Table S2. We found significant shifts in mean trait values in the home versus away ranges of invaders, but patterns were not consistent across growth habits or regions (Figure 4). For woody species, cell wall content and SLA of invaders changed significantly between France and the United States, but in opposite directions: Cell wall content was higher in the home range for invaders native to France, but lower in the home range for invaders native to the United States, with inverse relationships for SLA. Alkaloid concentrations were also higher in the home range for woody species native to the United States. For woody species native to Japan and invasive in the United States, leaf N was lower, and CN higher, in the home range. However, for all these changes, the variation across species in trait values was much larger than variance within species, with fixed effect (marginal) *R* ^2^ values of home–away models typically less than 0.2, except for cell wall content and SLA (Appendix S1: Table S3).

For herbaceous species, alkaloid concentrations were higher in the home range for herbaceous invaders from the United States, matching the pattern for woody species; these herbaceous invaders also had higher leaf C at home but lower fiber content (Figure 4). For herbaceous invaders native to France, leaf protein was greater in the home range. Overall, the effect size of home–away shifts for herbaceous species was lower than that for woody species, with intraspecific variation accounting for less than 16% of the total variation for all traits (Appendix S1: Table S2).

### Home–away shifts in the context of regional trait differences

**FIGURE 5:**
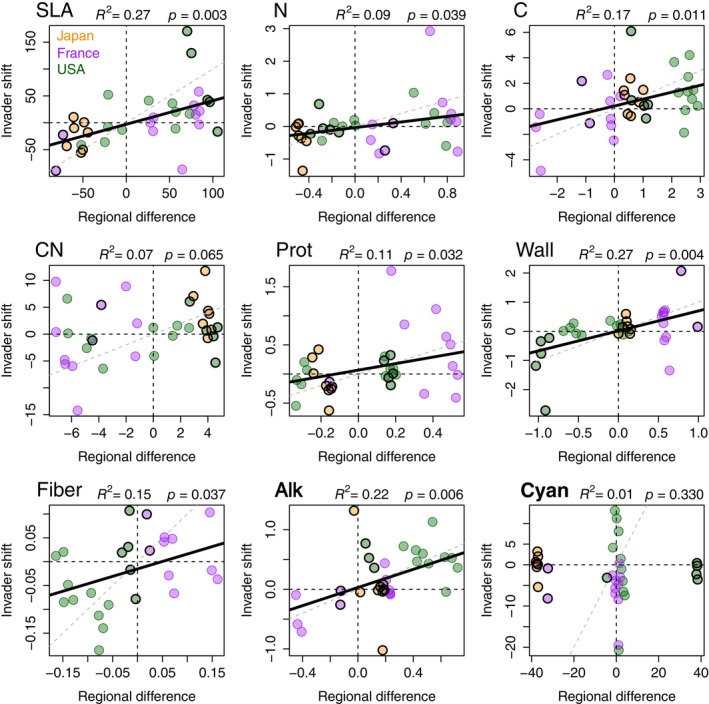
A test of the “join the locals” hypothesis for defense‐related traits across three regions. Each panel shows 33 paired values of each trait, with the Y axis showing the mean trait difference between the invader home and away regions (positive values indicate larger home range values), and the X axis showing the difference in mean native species values for the same regions (including only species of the same growth form, and excluding the focal invader species). Cyanogenic glycosides and alkaloids are considered qualitative defenses and labeled in bold, and the rest are quantitative. Points outlined in black indicate woody species. Black dashed lines show differences of 0 for each axis, and gray dashed lines indicate 1:1 relationships. Colors show native regions of each invader as in Figure 4. Ordinary least squares regression lines (with indicated *R* ^2^) are shown for non‐zero slopes along with bootstrapped *p* values. Traits are listed in Table 2. SLA, specific leaf area.

Appendix S1: Figures S1 and S2 show the distributions of all four populations (natives at home, invaders at home, invaders away, co‐occurring natives in the away range) for each trait and region of origin. For seven out of nine traits, the shift in mean trait values between the home and away ranges of invasive species was consistent with the overall trait difference for native species of those same regions (Figure 5). For CN, the relationship was positive but marginal, and we detected no relationship for cyanogenic glycosides (Figure 5). For all other traits, the home–away shift was significant and consistent with the regional difference for native species of the same growth form, and thus consistent with the “join the locals” hypothesis.

### Multivariate trait relationships

**FIGURE 6:**
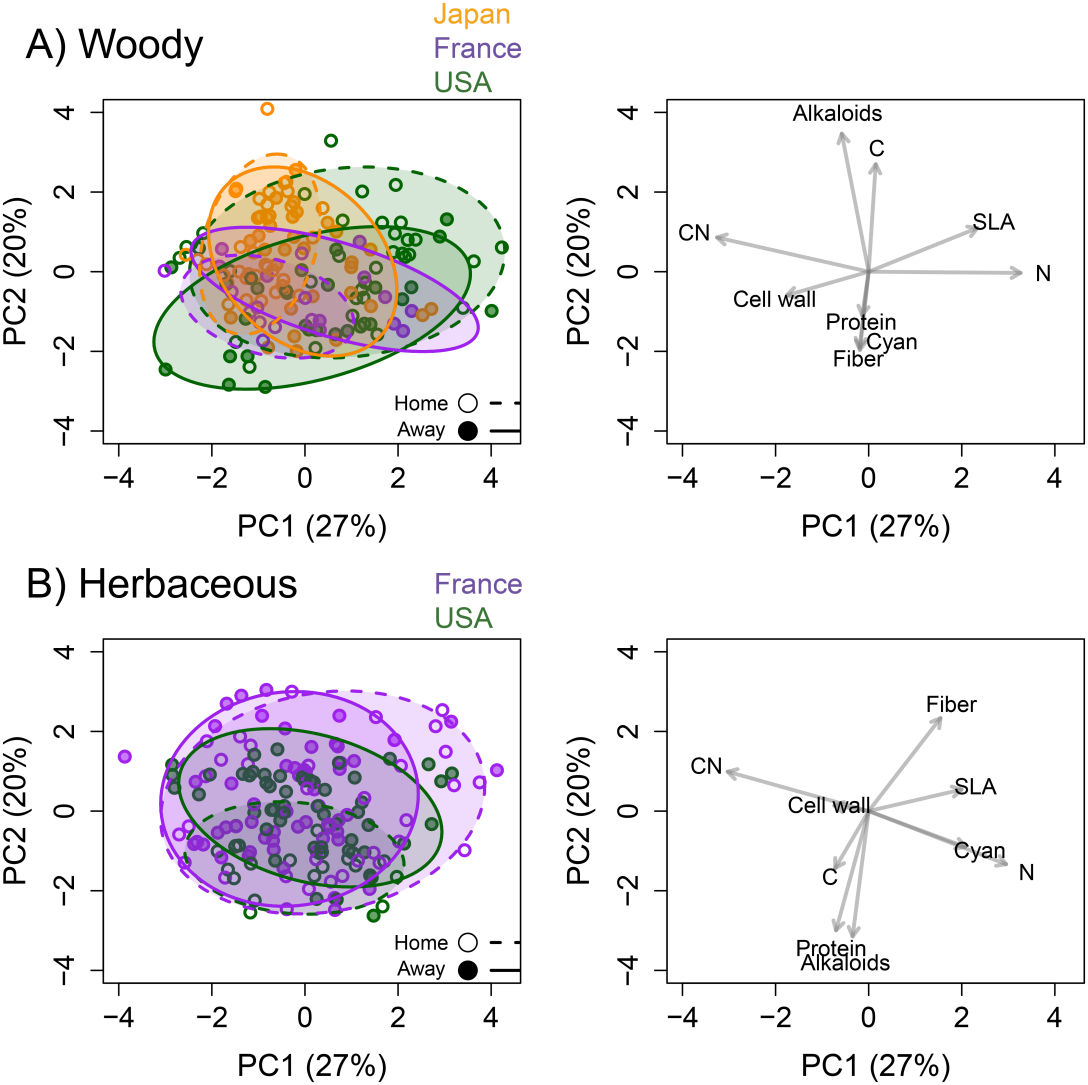
Multivariate patterns of leaf defensive traits, in separate analyses for (A) woody and (B) herbaceous species. Left panels show principal coordinate (PC) 1 and 2 scores for each sample, and right panels show corresponding loading of each trait along PCs 1 and 2. Colors for points and ellipses correspond to native ranges as in Figure 4; open symbols and dashed lines describe home populations, while closed symbols and solid lines indicate away‐range populations. Traits are listed in Table 2. In our study, there were no invasive herbs native to Japan. SLA, specific leaf area.

Defensive trait syndromes for woody invaders were most strongly characterized by variation in leaf N, CN, and SLA, loading on PC1 (eigenvalue = 0.27), with alkaloid concentration and leaf C forming a second principal axis of variation (eigenvalue = 0.20; eigenvalues for PCs 3 and 4 were 0.15 and 0.12, respectively) (Figure 6A). We found no association of home‐ versus away‐range values with PCs 1 or 2, but there was a strong effect of woody invader native region, where the mean of PC1 was higher for species native to the United States, and the mean of PC2 was lower for species native to France than that for the United States or Japan.

For herbaceous species of open habitats (Figure 6B), PC1 (eigenvalue = 0.27) was, as for woodies, driven by variation in leaf N, CN, and SLA, plus cyanogenic glycosides, while PC2 (eigenvalue = 0.20) was dominated by variation in alkaloid concentration and total proteins (PC3 and PC4 eigenvalues were 0.15 and 0.12, respectively). We found no relationship of invader PC1 scores with home versus away range, region, or native origin. However, there was a strong region effect for PC2, where PC2 values were significantly lower for US sites.

## DISCUSSION

In a cross‐continental survey of multiple species, we examined how leaf traits of woody and herbaceous invasive plants shift between home and away ranges, testing the EICAH and the SDH. We focused on whether quantitative traits (e.g., leaf N, C, CN, protein, fiber, and cell wall content) shift more than qualitative defensive traits (alkaloid or cyanogenic glycoside content) and tested a novel “join the locals” hypothesis (JTLH) whereby invaders adopt traits of co‐occurring native species in their away range. Our study revealed that home–away shifts in leaf defensive traits are common, typically shifting toward the regional mean of the recipient community. This pattern suggests that these defensive traits are part of a broader phenotype of leaf traits that are driven primarily by the physical environment, rather than release from enemies per se. Given the growing recognition that plant leaf traits are expressed as a set of syndromes reflecting economic constraints related to the uptake and use of a small number of limiting resources (Agrawal, 2020; Reich, 2014), and that such syndromes vary predictably according to site‐based resource supply rates (Chapin, 1980; Coley et al., 1985; Endara & Coley, 2011), we suggest the JTLH as a reasonable null model for home–away contrasts of functional traits in invasive plant species. Although this hypothesis is not mutually exclusive with EICAH or SDH for a single trait shift of a given invader, our use of reciprocal invasions in multiple habitat types across three geographic regions suggests that JTLH is a more general expectation of home–away trait shifts than those driven by enemy release alone. Our results thus add to the growing recognition of rapid local adaptation in invasive plants (Oduor et al., 2016) and place an imperative on understanding environmental differences between invader home and away ranges.

If the JTLH is based on environmental differences across home and away ranges, which environmental drivers were responsible for the trait shifts observed in our study? Despite our attempt to measure invaders in their home and away ranges across similar latitudes and habitat types, we found distinct soil, light, and climate regimes in our study regions. For example, sites in Japan were warmer and wetter than sites in France and the United States, and their associated low pH soils are likely the product of greater weathering rates (Huston, 2012). Although we did not measure soil N mineralization rates, the lower overall leaf N and protein content of leaves in Japan—the latter averaging only about two‐thirds that of leaves in France—may be the direct result of less fertile, more acidic soils (Table 3). Invaders native to Japan increased their leaf N and protein content in their away range, while those invaders native to France and the United States decreased these trait values in Japan (see Appendix S1: Figure S1), as expected from these soil differences. In addition, we found both native and invasive plants in France to have substantially greater cell wall mass (75% greater on average than those in Japan or the United States), which was associated with environmental differences that included both lower rainfall (roughly half that of Japan and the United States) and higher soil pH (7.3, more than 2 units higher than Japan sites). The cell wall–soil pH connection may be due to the availability of soil calcium; in non‐graminoid species, calcium pectate bonds in the cell wall can compensate for mechanical stability otherwise conferred by hemicellulose and cellulose (Xing et al., 2021). This may further explain why the cell wall mass of plants in France was high without concomitantly high values of leaf C (Table 2).

The greater SLA of native and invasive woody plants in the United States is likely due to darker forest conditions of the sites we sampled, particularly in comparison with Japan (on average 330 vs. 270 g m^−2^ SLA, and GLI values of 10% and 25%, respectively). However, it is unclear why plants in the United States expressed lower cyanogenic glycosides, but greater alkaloid concentrations, than plants in France or Japan. One possibility is that varying herbivore communities across regions induce different defensive metabolic pathways (Fuchs et al., 2017). Indeed, although we view environmental differences across regions as a common driver of JTLH, it is also possible that levels of generalist herbivory differ across regions in the absence of associated environmental variation, which may itself cause regional shifts in defense allocation. For example, mammalian herbivore communities (native and non‐native) have been strongly shaped by the human history of different biogeographic regions and have been shown to exert strong influence on leaf defensive strategies (O'Reilly‐Wapstra & Cowan, 2010). One weakness of our study is that we did not monitor the composition of herbivore communities in each region, and therefore cannot discriminate between environmental versus food web‐related drivers of JTLH.

To what extent do our results support changes in leaf traits from enemy release? The SDH suggests qualitative defenses against generalist herbivores, including cyanogenic glycosides and alkaloids, should increase relative to quantitative defenses in the away range of invaders (Huang et al., 2010; Müller‐Schärer et al., 2004). However, we observed a strong decrease in alkaloid concentration for invaders from the United States in their away ranges in both France and Japan, and comparatively little change in the expression of cyanogenic glycosides for any invasive group. Perhaps most telling were levels of the latter for *P. serotina*, a European invader that is native to the United States and can produce toxic cyanogenic glycosides such as prunasin in large quantities (as much as 6% leaf dry weight; Santos Pimenta et al., 2014). This species seems to be equally toxic in its home and away ranges (see also Halarewicz & Gabryś, 2012). We also found *P. serotina* to have two orders of magnitude larger concentration of cyanogenic glycosides than most other species in our analysis, which suggests that most of our focal species do not generally invest in this group of compounds for defense, although levels were significantly higher in herbaceous species. For quantitative defensive traits, we found some home–away shifts that are consistent with the EICAH. For example, woody invaders from France reduced their cell wall content in their invasive range in the United States. However, we observed the opposite shift in this trait in woody invaders from the United States to France, and herbaceous invaders from the United States increased their fiber concentration in their away ranges, in opposition to the EICAH. For most other leaf traits, we saw no consistent directional shift between home and away ranges. Instead, changes reflected differences in the regional means of native species. Overall, our results suggest that enemy release is not the primary driver of observed leaf trait shifts in invasive species (Felker‐Quinn et al., 2013). Nonetheless, a more definitive test would include estimates of generalist and specialist herbivory rates on native and invasive plants across regions, which would exclude the possibility that loss of specialist enemies covaries with environmental differences.

Many researchers have argued that woody species are more subject to herbivore pressure based on greater apparency; that is, they are larger and longer‐lived than herbaceous species, and thus incur lower foraging costs for herbivores (Feeny, 1976; Martini et al., 2021; Rhoades & Cates, 1976; Strauss et al., 2015). Furthermore, Smilanich et al. (2016) showed that woody species are twice as likely to express quantitative defensive traits than herbaceous species, while the latter are three times as likely to express qualitative defenses. Our results are broadly consistent with this pattern: Herbaceous species expressed higher levels of the qualitative cyanogenic glycoside defenses, while the only two quantitative defensive traits that systematically differed between growth forms were leaf C and fiber content, and both were higher for woody species. We acknowledge that our study design limits our ability to test apparency theory per se, given woody and herbaceous species were also found in different habitat types that varied in resource availability, in addition to other differences in allocation strategies between growth forms. Nonetheless, greater overall allocation to a defensive trait in the home range of an invader suggests a larger capacity for downregulation in its away range, particularly if such reallocation comes at a larger potential gain to photosynthetic and growth capacity, as predicted by the EICAH and growth‐defense theory (Coley et al., 1985). On the other hand, the SDH suggests quantitative defense traits are less likely to shift between invader home and away ranges (Müller‐Schärer et al., 2004), which may suggest trait shifts induced by enemy release are more frequent in herbaceous invaders given their greater investment in qualitative defense. Our results indicate that home–away shifts in defense‐related traits are common in both woody and herbaceous species, but in neither do they conform to predictions of the EICAH or the SDH. We suggest this is due in part to the role of regional environmental differences, but we also point out that some aspects of the SDH may be based on unfounded assumptions. For example, the meta‐analysis of Smilanich et al. (2016) found that, counter to predictions from apparency theory, qualitative defenses were more effective against specialist than generalist herbivores. This calls into question whether particular leaf traits can be reliably classified into specialist versus generalist defenses and suggests the SDH is better addressed by quantifying rates of herbivory from consumers of known diet breadths, rather than through particular plant traits per se (Zhang et al., 2018; A. Ameline et al., unpublished manuscript).

Our study is unusual in considering both quantitative and qualitative defensive traits for many species, offering an opportunity to assess levels of coordination in leaf traits related to defensive syndromes (Agrawal & Fishbein, 2006). As expected from plant economics theory (Díaz et al., 2016), for both woody and herbaceous species we identified a primary axis of trait variation separating N‐rich, high SLA species from those of high CN and low SLA (Figure 6). However, the secondary axis did not reflect a trade‐off between qualitative and quantitative traits, nor did it show a consistent relationship for plant investments in structural (cell wall, fiber content) versus nutritional (protein content) traits. Meta‐analyses have also failed to find evidence of plant defense syndromes (Koricheva et al., 2004; Moles et al., 2013), although we caution that any such study is limited by the relatively small number of traits considered in relation to the myriad attributes under selection related to herbivory (Agrawal, 2007). Furthermore, we did not detect a shift in the multivariate expression of defense traits between the home and away ranges of invaders for any region or growth form. This contrasts with Liu et al. (2021), who found away‐range populations of an invasive herb to express a distinct set of growth‐defense traits compared to its home range. Our focus on multiple species in the present study did not allow sufficient sample sizes to test for trade‐offs or syndromes within particular invasive species. However, in a companion study of 38 populations of *Reynoutria japonica* across the same regions, we also failed to find evidence of a simple home–away shift in growth‐defense strategies, or trade‐offs involving allocation to different types of defenses (Griffin‐Nolan et al., 2024). As argued by Ni et al. (2020), we suspect that a more general trend is that many herbaceous invaders express high photosynthetic rates without loss of toxic compounds in the away range, because both behaviors are driven by local resource supply rates rather than herbivore load per se. Furthermore, there is evidence that the competitive advantage of woody invaders in forested habitats may be a more direct result of reduced consumption in the away range rather than resource allocation away from defense (Fridley et al., 2023). This does not rule out other forms of resource reallocation in the away range. For example, Heberling et al. (2016) found large shifts in leaf N resorption and PNUE between the home and away ranges of two woody species in France and the United States, and Martinez et al. (2019) found evidence for changes in N allocation between photosynthetic functions for woody invaders between the United States and Japan.

## CONCLUSION

In a study of 27 invasive species of field and forest habitats across their home and away ranges in three temperate regions, we found little support for invasion hypotheses that assume shifts in resource allocation at the leaf level are driven by enemy release. Our principal finding is that shifts in leaf traits are common between home and away ranges, but that such shifts are predicted by the traits of the local native community, which are most likely driven by environmental factors controlling resource supply rates. We suggest this “join the locals” pattern of home–away trait shifts as a null model for studies of adaptive evolution in invasive species, which motivates trait comparisons across four separate populations, including those of the invader at home and away, as well as native communities of the invader both at home and in the away range. Although hypotheses that invoke resource reallocation toward competitiveness due to enemy release remain a common explanation for invader success, to date supporting evidence remains scarce. Furthermore, related hypotheses such as the EICAH and the SDH will remain difficult to test in the absence of a solid framework of plant growth‐defense trade‐offs, if indeed such trade‐offs are common.

## AUTHOR CONTRIBUTIONS

This study was conceived by Jason Fridley with input from co‐PIs Guillaume Decocq, Thomas Kichey, and Kouki Hikosaka. Data collection was led by Robert J. Griffin‐Nolan, with significant contributions by Lamine Bensaddek, Thomas Kichey, Julie LeVonne, and Masako Mishio. Data analysis was performed by Jason Fridley. The initial draft of the manuscript was written by Jason Fridley and Robert J. Griffin‐Nolan. All authors read and approved the final manuscript.

## CONFLICT OF INTEREST STATEMENT

The authors declare no conflicts of interest.

## Supporting information


Appendix S1.

## Data Availability

Data and R code (Fridley, 2024) are available in Zenodo at https://doi.org/10.5281/zenodo.13362541.
