# Supplementary material for: Joining the locals: Plant invaders shift leaf defenses to match native neighbors
Source: Ecology. 2025 Jun 8;106(6):e70129. doi: 10.1002/ecy.70129 (PMC12145798; doi:10.1002/ecy.70129)
Supplement: Supplementary file 1 — Appendix S1. [file ECY-106-e70129-s001.pdf]

Appendix S1

Authors: Jason Fridley, Robert J. Griffin-Nolan, Lamine Bensaddek, Guillaume Decocq, Kouki Hikosaka, Thomas Kichey, Julie LeVonne, Masako Mishio

Article Title: Joining the locals: Plant invaders shift leaf defenses to match native neighbors

Journal: Ecology

Table S1. P values along with associated t statistics and degrees of freedom (df) for Student's t tests concerning the null hypothesis that trait values do not different between woody and herbaceous species (Figure 3).

**Trait**	**t**	**df**	**P value**
Cyan	8.5589	318.54	<0.0001
Alkaloids	0.0113	378.32	0.991
Total fiber	-5.3562	316.86	<0.0001
Cell wall mass	-1.1157	300.56	0.2654
Total protein	1.3821	322.86	0.1679
CN	-1.0861	360.22	0.2782
Cmass	-3.7537	347.09	0.0002
Nmass	1.4930	363.15	0.3163
SLA	-5.7690	335.15	<0.0001

Table S2. Mean (+/- 1 S.E.) trait values for home and away populations of each species. Sample sizes are listed in Table 1. Non-invasive species were only sampled in their home ranges. ND = no data. Units are described in Table 2. For species with multiple away ranges, all away range populations are included in the Away column statistics.

		**SLA**		**Leaf N**		**Leaf C**		**Leaf CN**	
**Group**	**Species**	**Home**	**Away**	**Home**	**Away**	**Home**	**Away**	**Home**	**Away**
Woody	*Acer negundo*	284 ± 8.6	245 ± 31	2.85 ± 0.11	2.17 ± 0.20	46.7 ± 2.3	44.4 ± 0.94	16.5 ± 0.83	21.2 ± 2.2
	Acer pseudoplatanus	229 ± 35		2.26 ± 0.15		45.0 ± 0.27		20.2 ± 1.1	
	Berberis thunbergii	334 ± 26	324 ± 23	1.90 ± 0.044	1.81 ± 0.11	46.9 ± 0.26	45.8 ± 0.68	24.8 ± 0.51	25.6 ± 1.4
	Celastrus orbiculatus	331 ± 32	341 ± 33	1.76 ± 0.10	2.12 ± 0.24	43.0 ± 0.46	41.5 ± 0.47	24.7 ± 1.4	20.3 ± 2.4
	Euonymus alatus	235 ± 11	253 ± 29	1.90 ± 0.071	2.19 ± 0.21	45.7 ± 0.43	43.3 ± 0.51	24.2 ± 1	20.4 ± 2
	Humulus lupulus	419 ± 53		3.25 ± 0.39		38.5 ± 2.6		12.2 ± 1.1	
	Laburnum anagyroides	289 ± 37		2.73 ± 0.22		45.1 ± 0.61		17.0 ± 1.1	
	Lindera benzoin	485 ± 41		2.80 ± 0.18		43.7 ± 1.0		15.7 ± 0.7	
	Lonicera canadensis	323 ± 16		1.29 ± 0.11		43.4 ± 0.71		34.7 ± 3.6	
	Lonicera japonica	282 ± 29	338 ± 7.3	2.04 ± 0.11	2.06 ± 0.12	47.1 ± 0.31	45.7 ± 0.51	23.2 ± 1.3	22.4 ± 1.1
	Lonicera morrowii	261 ± 17	261 ± 1.2	1.93 ± 0.24	1.86 ± 0.14	54.3 ± 8.7	46.2 ± 2.1	27.9 ± 1.1	25.1 ± 1.7
	Parthenocissus quinquefolia	466 ± 49	295 ± 29	2.61 ± 0.19	2.67 ± 0.29	43.7 ± 0.51	43.3 ± 0.3	16.9 ± 1.1	17.3 ± 1.8
	Prunus avium	202 ± 20	225 ± 23	2.03 ± 0.18	1.93 ± 0.2	43.3 ± 0.5	44.4 ± 0.073	22.3 ± 2.2	23.5 ± 2.2
	Prunus serotina	301 ± 22	259 ± 29	2.19 ± 0.14	2.29 ± 0.15	44.1 ± 0.21	44.9 ± 1.1	20.5 ± 1.4	19.8 ± 0.95
	Quercus robur	227 ± 20		2.48 ± 0.19		47.1 ± 0.65		19.4 ± 1.7	
	Quercus rubra	220 ± 11	236 ± 18	1.64 ± 0.034	1.83 ± 0.19	47.8 ± 0.38	41.7 ± 3.9	29.1 ± 0.66	23 ± 1.1
	Rhamnus cathartica	244 ± 15	333 ± 14	2.39 ± 0.17	3.13 ± 0.034	44.7 ± 1.3	42.5 ± 0.48	19.0 ± 1.2	13.6 ± 0.18
	Robinia pseudo-acacia	431 ± 38	302 ± 28	3.37 ± 0.25	3.60 ± 0.32	47.7 ± 1.6	47.0 ± 0.69	14.8 ± 1.5	13.5 ± 1.2
	Rosa multiflora	382 ± 15	425 ± 29	1.69 ± 0.066	3.06 ± 0.005	43.9 ± 0.72	43.4 ± 0.005	26.0 ± 0.62	14.2 ± 0.022
	Viburnum acerifolium	328 ± 15		1.58 ± 0.12		44.3 ± 0.45		28.6 ± 2.1	
	Viburnum dilatatum	271 ± 12	321 ± 29	1.49 ± 0.059	1.94 ± 0.061	46.3 ± 0.44	46.7 ± 0.42	31.2 ± 1.3	24.1 ± 0.67

Table S2 (con't)

		SLA		Leaf N		Leaf C		Leaf CN	
**Group**	**Species**	**Home**	**Away**	**Home**	**Away**	**Home**	**Away**	**Home**	**Away**
Herbaceous	*Agrostis gigantea*	274 ± 36	241 ± 30	2.21 ± 0.56	1.82 ± 0.23	42.8 ± 0.6	43.6 ± 0.2	20.8 ± 5.5	25.6 ± 3.3
	Agrostis stolonifera	449 ± 18	948 ± 250	3.18 ± 0.21	2.44 ± 0.28	41.5 ± 1.8	43.9 ± 1.9	13.3 ± 1.2	19.3 ± 3
	Ambrosia artemisiifolia	251 ± 0	251 ± 30	2.86 ± 0.47	2.76 ± 0.24	45.6 ± 3.9	43.3 ± 0.94	17.1 ± 3.8	16.6 ± 1.5
	Anthoxanthum odoratum	247 ± 11	232 ± 25	1.61 ± 0.20	1.46 ± 0.066	44.3 ± 0.44	41.6 ± 1.6	29.0 ± 3.1	28.6 ± 1.6
	Artemisia vulgaris	237 ± 27	237 ± 0	2.44 ± 0.11	2.86 ± 0.21	44.8 ± 0.53	46.2 ± 0.82	18.5 ± 0.77	16.5 ± 1.4
	Bidens frondosa	361 ± 0	361 ± 20	3.27 ± 0.20	2.89 ± 0.33	44.5 ± 0.21	43.5 ± 0.58	13.6 ± 0.77	16.7 ± 1.8
	Bidens tripartita	379 ± 54		3.75 ± 0.53		44.0 ± 1.3		12.2 ± 1.5	
	Chenopodium album	202 ± 16	144 ± 6.9	4.60 ± 0.23	1.68 ± 0.13	38.5 ± 0.78	37.5 ± 0.93	8.5 ± 0.6	22.7 ± 1.7
	Cirsium japonicum	113 ± 8.6		1.60 ± 0.24		41.9 ± 1.3		28.4 ± 4.7	
	Conyza canadensis	236 ± 13	198 ± 7.1	2.56 ± 0.27	2.37 ± 0.21	45.2 ± 0.5	44.2 ± 0.21	18.4 ± 1.8	19.8 ± 1.4
	Dactylis glomerata	292 ± 24		2.72 ± 0.16		44.8 ± 0.65		16.7 ± 1.2	
	Daucus carota	325 ± 34		2.35 ± 0.11		43.2 ± 0.69		18.5 ± 0.94	
	Erigeron annuus	237 ± 9.1	246 ± 17	2.10 ± 0.34	2.03 ± 0.32	47.1 ± 1.5	44.7 ± 0.75	23.9 ± 3.2	25.9 ± 3.1
	Eupatorium cannabinum	340 ± 42		3.27 ± 0.33		48.9 ± 3.7		15.5 ± 1.7	
	Eupatorium glehnii	171 ± 8.4		2.46 ± 0.23		45.3 ± 0.47		19.0 ± 1.6	
	Leucanthemum vulgaris	195 ± 12	202 ± 11	1.49 ± 0.076	2.28 ± 0.15	43.1 ± 1.1	42.8 ± 0.43	29.1 ± 1.3	19.6 ± 1.1
	Plantago asiatica	171 ± 13		1.44 ± 0.21		40.2 ± 0.66		29.6 ± 3.2	
	Plantago lanceolata	188 ± 21	173 ± 10	2.26 ± 0.26	2.10 ± 0.3	42.6 ± 0.34	45.2 ± 0.92	20.1 ± 2.2	25.2 ± 4
	Senecio jacobaea	180 ± 15		2.23 ± 0.09		44.0 ± 0.29		19.9 ± 0.87	
	Senecio vulgaris	274 ± 46		3.92 ± 0.35		40.2 ± 1.3		10.6 ± 1.1	
	Solidago gigantea	172 ± 0	172 ± 14	2.15 ± 0.25	2.30 ± 0.23	51.3 ± 5.6	50.5 ± 5.1	24.0 ± 0.47	20.2 ± 2.4
	Solidago virgaurea	228 ± 23		1.83 ± 0.1		44.8 ± 0.78		24.9 ± 1.3	
	Youngia japonica	340 ± 25		2.18 ± 0.4		42.5 ± 1.5		20.9 ± 4	

Table S2 (con't)

		**Total Protein**		**Cell wall mass**		**Total Fiber**	
**Group**	**Species**	**Home**	**Away**	**Home**	**Away**	**Home**	**Away**
Woody	*Acer negundo*	0.770 ± 0.062	0.445 ± 0.07	0.621 ± 0.10	3.34 ± 0.5	0.365 ± 0.016	0.444 ± 0.026
	Acer pseudoplatanus	0.763 ± 0.12		1.450 ± 0.53		0.509 ± 0.032	
	Berberis thunbergii	0.338 ± 0.022	0.555 ± 0.067	0.544 ± 0.11	0.628 ± 0.037	0.396 ± 0.0068	0.415 ± 0.009
	Celastrus orbiculatus	0.529 ± 0.11	0.732 ± 0.22	0.796 ± 0.076	0.454 ± 0.039	ND	0.524 ± 0.018
	Euonymus alatus	0.935 ± 0.16	0.926 ± 0.16	1.41 ± 0.13	1.48 ± 0.36	ND	0.455 ± 0.042
	Humulus lupulus	0.554 ± 0.19		2.97 ± 0.98		0.454 ± 0.03	
	Laburnum anagyroides	0.400 ± 0.06		1.01 ± 0.23		0.486 ± 0.029	
	Lindera benzoin	0.915 ± 0.27		0.300 ± 0.03		0.501 ± 0.017	
	Lonicera canadensis	0.896 ± 0.3		0.749 ± 0.11		0.445 ± 0.026	
	Lonicera japonica	0.774 ± 0.18	0.352 ± 0.027	0.731 ± 0.16	0.566 ± 0.048	ND	0.352 ± 0.029
	Lonicera morrowii	0.436 ± 0.054	1.06 ± 0.24	0.486 ± 0.08	0.410 ± 0.08	0.230 ± NA	0.326 ± 0.025
	Parthenocissus quinquefolia	0.670 ± 0.12	0.661 ± 0.19	0.288 ± 0.04	0.514 ± 0.24	0.471 ± 0.025	0.440 ± 0.043
	Prunus avium	0.609 ± 0.1	0.745 ± 0.14	1.39 ± 0.43	1.23 ± 0.17	0.442 ± 0.043	0.419 ± 0.043
	Prunus serotina	1.01 ± 0.13	0.778 ± 0.16	0.995 ± 0.44	1.76 ± 0.67	0.440 ± 0.019	0.457 ± 0.036
	Quercus robur	1.06 ± 0.29		0.831 ± 0.3		0.508 ± 0.048	
	Quercus rubra	0.757 ± 0.075	0.949 ± 0.17	1.46 ± 0.22	2.64 ± 0.52	0.559 ± 0.0034	0.540 ± 0.036
	Rhamnus cathartica	0.564 ± 0.055	0.817 ± 0.12	2.42 ± 0.48	0.343 ± 0.11	0.472 ± 0.030	0.373 ± 0.049
	Robinia pseudo-acacia	0.841 ± 0.14	0.905 ± 0.26	1.02 ± 0.26	1.36 ± 0.36	0.453 ± 0.028	0.346 ± 0.056
	Rosa multiflora	0.447 ± 0.097	0.725 ± 0.097	0.847 ± 0.11	0.252 ± 0.04	ND	0.441 ± 0.026
	Viburnum acerifolium	0.726 ± 0.088		0.553 ± 0.072		0.455 ± 0.021	
	Viburnum dilatatum	0.973 ± 0.13	0.688 ± 0.075	1.08 ± 0.098	0.94 ± 0.085	ND	0.431 ± 0.0097

Table S2 (con't)

		Total Protein		Cell wall mass		Total Fiber	
**Group**	**Species**	**Home**	**Away**	**Home**	**Away**	**Home**	**Away**
Herbaceous	*Agrostis gigantea*	1.13 ± 0.51	0.489 ± 0.044	1.41 ± 0.39	1.72 ± 0.27	0.648 ± NA	0.606 ± 0.019
	Agrostis stolonifera	0.338 ± 0.029	0.354 ± 0.05	1.52 ± 0.7	0.86 ± 0.36	0.634 ± 0.023	0.582 ± 0.026
	Ambrosia artemisiifolia	0.863 ± 0.065	0.985 ± 0.2	0.529 ± 0.2	0.624 ± 0.12	0.248 ± 0.028	0.337 ± 0.022
	Anthoxanthum odoratum	0.828 ± 0.22	0.321 ± 0.068	0.569 ± 0.14	1.91 ± 0.47	0.522 ± 0.030	0.551 ± 0.041
	Artemisia vulgaris	1.72 ± 0.35	0.981 ± 0.19	0.917 ± 0.07	1.11 ± 0.19	0.377 ± 0.010	0.395 ± 0.029
	Bidens frondosa	0.745 ± 0.059	0.790 ± 0.17	0.255 ± 0.042	0.32 ± 0.068	0.281 ± 0.100	0.429 ± 0.033
	Bidens tripartita	0.408 ± 0.09		1.4 ± 1.2		0.387 ± 0.041	
	Chenopodium album	0.684 ± 0.067	0.546 ± 0.024	1.56 ± 0.41	1.28 ± 0.31	0.384 ± 0.045	0.451 ± 0.025
	Cirsium japonicum	0.860 ± 0.074		1.02 ± 0.15		0.372 ± 0.050	
	Conyza canadensis	0.624 ± 0.058	0.597 ± 0.074	0.834 ± 0.17	0.679 ± 0.18	0.345	0.368 ± 0.02
	Dactylis glomerata	1.38 ± 0.16		1.42 ± 0.21		0.605 ± 0.021	
	Daucus carota	2.02 ± 0.79		0.837 ± 0.1		0.220 ± 0.011	
	Erigeron annuus	0.682 ± 0.11	0.532 ± 0.06	0.658 ± 0.11	0.401 ± 0.067	0.205 ± 0.010	0.291 ± 0.014
	Eupatorium cannabinum	0.654 ± 0.055		1.67 ± 0.79		0.416 ± 0.033	
	Eupatorium glehnii	0.657 ± 0.096		0.479 ± 0.049		0.325 ± 0.015	
	Leucanthemum vulgaris	1.84 ± 0.75	0.813 ± 0.075	1.32 ± 0.13	0.687 ± 0.057	0.225 ± 0.0091	0.261 ± 0.018
	Plantago asiatica	0.450 ± 0.05		0.976 ± 0.073		0.430 ± 0.033	
	Plantago lanceolata	0.459 ± 0.023	0.829 ± 0.055	1.61 ± 0.44	1.02 ± 0.1	0.428 ± 0.020	0.355 ± 0.017
	Senecio jacobaea	0.522 ± 0.086		1.00 ± 0.39		0.305 ± 0.028	
	Senecio vulgaris	0.435 ± 0.048		1.30 ± 0.38		0.287 ± 0.019	
	Solidago gigantea	0.845 ± 0.068	0.914 ± 0.14	1.17 ± 0.16	0.955 ± 0.18	0.284 ± 0.0052	0.313 ± 0.031
	Solidago virgaurea	0.900 ± 0.35		0.789 ± 0.22		0.355 ± 0.028	
	Youngia japonica	0.394 ± 0.048		0.296 ± 0.041		0.297 ± 0.017	

Table S2 (con't)

		Alkaloids		Cyanogenic glycosides	
**Group**	**Species**	**Home**	**Away**	**Home**	**Away**
Woody	*Acer negundo*	0.777 ± 0.26	0.250 ± 0.062	7.01 ± 0.96	7.23 ± 1.2
	Acer pseudoplatanus	0.252 ± 0.081		10.2 ± 1.3	
	Berberis thunbergii	1.42 ± 0.18	0.098 ± 0.022	10.0 ± 0.76	10.6 ± 1.8
	Celastrus orbiculatus	0.104 ± 0.021	0.118 ± 0.022	9.51 ± 1.8	9.24 ± 1.5
	Euonymus alatus	0.150 ± 0.031	0.072 ± 0.030	8.30 ± 1.1	5.08 ± 1.5
	Humulus lupulus	0.177 ± 0.032		13.9 ± 2.9	
	Laburnum anagyroides	0.552 ± 0.35		21.5 ± 4.8	
	Lindera benzoin	0.241 ± 0.12		13.5 ± 3.3	
	Lonicera canadensis	0.136 ± 0.06		8.21 ± 0.35	
	Lonicera japonica	0.377 ± 0.049	0.392 ± 0.092	6.58 ± 0.6	12 ± 1.2
	Lonicera morrowii	1.15 ± 0.44	1.20 ± 0.082	7.09 ± 0.34	6.99 ± 0.79
	Parthenocissus quinquefolia	0.091 ± 0.055	0.129 ± 0.042	7.67 ± 0.023	7.25 ± 0.77
	Prunus avium	0.190 ± 0.053	0.449 ± 0.15	11.8 ± 2.1	12.6 ± 0.54
	Prunus serotina	0.207 ± 0.066	0.164 ± 0.038	305 ± 3.9	308 ± 4.1
	Quercus robur	0.101 ± 0.018		10.4 ± 0.83	
	Quercus rubra	0.951 ± 0.24	0.181 ± 0.052	9.36 ± 1.7	11.7 ± 5.8
	Rhamnus cathartica	0.174 ± 0.058	0.199 ± 0.079	12.2 ± 2.3	20.4 ± 2.5
	Robinia pseudo-acacia	0.511 ± 0.097	0.150 ± 0.058	5.55 ± 1.4	9.11 ± 2.3
	Rosa multiflora	0.142 ± 0.026	0.142 ± 0.044	12.6 ± 3.2	12 ± 3.5
	Viburnum acerifolium	0.111 ± 0.018		11.3 ± 3.9	
	Viburnum dilatatum	0.178 ± 0.032	1.20 ± 0.17	6.45 ± 0.66	4.43 ± 0.39

Table S2 (con't)

		Alkaloids		Cyanogenic glycosides	
**Group**	**Species**	**Home**	**Away**	**Home**	**Away**
Herbaceous	*Agrostis gigantea*	0.597 ± 0.300	0.160 ± 0.031	16.4 ± 5.6	21.1 ± 6.3
	Agrostis stolonifera	0.083 ± 0.021	0.168 ± 0.046	16.6 ± 3.1	36.1 ± 11
	Ambrosia artemisiifolia	1.24 ± 0.35	0.338 ± 0.13	18.1 ± 1.2	29.0 ± 4.8
	Anthoxanthum odoratum	0.175 ± 0.054	0.190 ± 0.077	16.9 ± 3.2	25.3 ± 11
	Artemisia vulgaris	0.629 ± 0.056	0.722 ± 0.24	26.6 ± 3.9	29.2 ± 1.9
	Bidens frondosa	0.853 ± 0.29	0.641 ± 0.34	29.3 ± 2.1	20.6 ± 2.5
	Bidens tripartita	0.480 ± 0.34		12.8 ± 1.2	
	Chenopodium album	0.108 ± 0.027	0.174 ± 0.05	35.7 ± 8.3	41.4 ± 10
	Cirsium japonicum	0.224 ± 0.07		23.4 ± 5.7	
	Conyza canadensis	0.518 ± 0.27	0.118 ± 0.022	22 ± 2.3	12.2 ± 1.8
	Dactylis glomerata	1.61 ± 0.33		25.9 ± 3	
	Daucus carota	0.524 ± 0.14		23 ± 9.8	
	Erigeron annuus	0.685 ± 0.19	0.123 ± 0.022	10.6 ± 0.51	16.3 ± 1.9
	Eupatorium cannabinum	0.096 ± 0.032		10.1 ± 2	
	Eupatorium glehnii	0.260 ± 0.06		15.6 ± 0.82	
	Leucanthemum vulgaris	0.473 ± 0.13	0.526 ± 0.12	10.3 ± 0.98	12.0 ± 1.2
	Plantago asiatica	0.125 ± 0.015		5.52 ± 0.68	
	Plantago lanceolata	0.088 ± 0.016	0.443 ± 0.14	5.87 ± 0.46	9.24 ± 1.1
	Senecio jacobaea	0.238 ± 0.057		16.8 ± 1.1	
	Senecio vulgaris	0.266 ± 0.066		17.4 ± 5.2	
	Solidago gigantea	0.890 ± 0.19	0.284 ± 0.069	13.1 ± 1.2	16.6 ± 2.3
	Solidago virgaurea	0.243 ± 0.074		18.5 ± 3.4	
	Youngia japonica	0.185 ± 0.15		16.1	

Table S3. Marginal (fixed effects) and conditional (fixed and random effects) R^2 values of hierarchical models of home-away shifts for each trait, separated by woody and herbaceous groups.

	**Woody**		**Herbaceous**	
**Trait**	**Marginal R^2**	**Conditional R^2**	**Marginal R^2**	**Conditional R^2**
SLA	0.31	0.45	0.04	0.44
N	0.09	0.57	0.03	0.22
C	0.04	0.15	0.11	0.27
CN	0.17	0.57	0.02	0.23
Protein	0.03	0.11	0.1	0.27
Cell wall	0.38	0.42	0.02	0.22
Fiber	0.06	0.25	0.16	0.78
Alkaloids	0.10	0.37	0.14	0.30
Cyan	0.03	0.27	0.03	0.33

Table S4. Tukey Honest Significant Difference (HSD) intervals and associated P values for post-hoc tests of pairwise regional differences in leaf traits. Trait units and ANOVA statistics are listed in Table 2.

Trait	Contrast	Differenc	Interval lower range	Interval upper range	P value
Cyan	France-USA	0.2296	0.0401	0.4190	0.0127
Cyan	Japan-USA	0.1822	-0.0209	0.3854	0.0890
Cyan	Japan-France	-0.0473	-0.2387	0.1440	0.8298
Alk	France-USA	-0.6537	-0.9496	-0.3578	<0.001
Alk	Japan-USA	-0.4336	-0.7551	-0.1120	0.0046
Alk	Japan-France	0.2201	-0.0868	0.5271	0.2113
Fiber	France-USA	0.0187	-0.0152	0.0526	0.3976
Fiber	Japan-USA	0.0237	-0.0159	0.0634	0.3371
Fiber	Japan-France	0.0050	-0.0326	0.0427	0.9468
Cell wall	France-USA	0.4067	0.1804	0.6329	<0.001
Cell wall	Japan-USA	0.1533	-0.0855	0.3921	0.2869
Cell wall	Japan-France	-0.2534	-0.4929	-0.0138	0.0353
Protein	France-USA	-0.0916	-0.2452	0.0620	0.3406
Protein	Japan-USA	-0.2824	-0.4449	-0.1198	0.0002
Protein	Japan-France	-0.1908	-0.3512	-0.0304	0.0149
CN	France-USA	-0.1352	-0.2283	-0.0422	0.0020
CN	Japan-USA	0.1223	0.0230	0.2217	0.0111
CN	Japan-France	0.2576	0.1663	0.3489	<0.001
C%	France-USA	-0.0356	-0.0560	-0.0152	0.0001
C%	Japan-USA	-0.0249	-0.0467	-0.0031	0.0202
C%	Japan-France	0.0107	-0.0093	0.0307	0.4196
N%	France-USA	0.0908	-0.0006	0.1823	0.0521
N%	Japan-USA	-0.1513	-0.2491	-0.0535	0.0009
N%	Japan-France	-0.2421	-0.3321	-0.1521	<0.001
SLA	France-USA	-0.2231	-0.3328	-0.1135	<0.001
SLA	Japan-USA	-0.3179	-0.4332	-0.2026	<0.001
SLA	Japan-France	-0.0948	-0.1936	0.0041	0.0635

Table S5. Tukey Honest Significant Difference (HSD) intervals and associated P values for post-hoc tests of pairwise regional differences in site environmental characteristics. Variable units and ANOVA statistics are listed in Table 3.

**Variable**	**Contrast**	**Difference**	**Interval lower rang**	**Interval upper ran**	**P value**
Soil pH	France-ENA	1.1323	0.7097	1.5550	<0.001
Soil pH	Japan-ENA	-1.0480	-1.5451	-0.5509	<0.001
Soil pH	Japan-France	-2.1803	-2.6278	-1.7327	<0.001
GLI	France-ENA	8.9357	-2.6254	20.4968	0.1546
GLI	Japan-ENA	14.5925	4.0824	25.1026	0.0050
GLI	Japan-France	5.6568	-6.4640	17.7776	0.4913
MAT	France-ENA	2.0589	1.5257	2.5922	<0.001
MAT	Japan-ENA	2.4709	1.8260	3.1158	<0.001
MAT	Japan-France	0.4119	-0.1943	1.0181	0.2442
MAP	France-ENA	-380.6942	-413.5056	-347.8828	<0.001
MAP	Japan-ENA	227.7051	188.0214	267.3888	<0.001
MAP	Japan-France	608.3993	571.0958	645.7028	<0.001

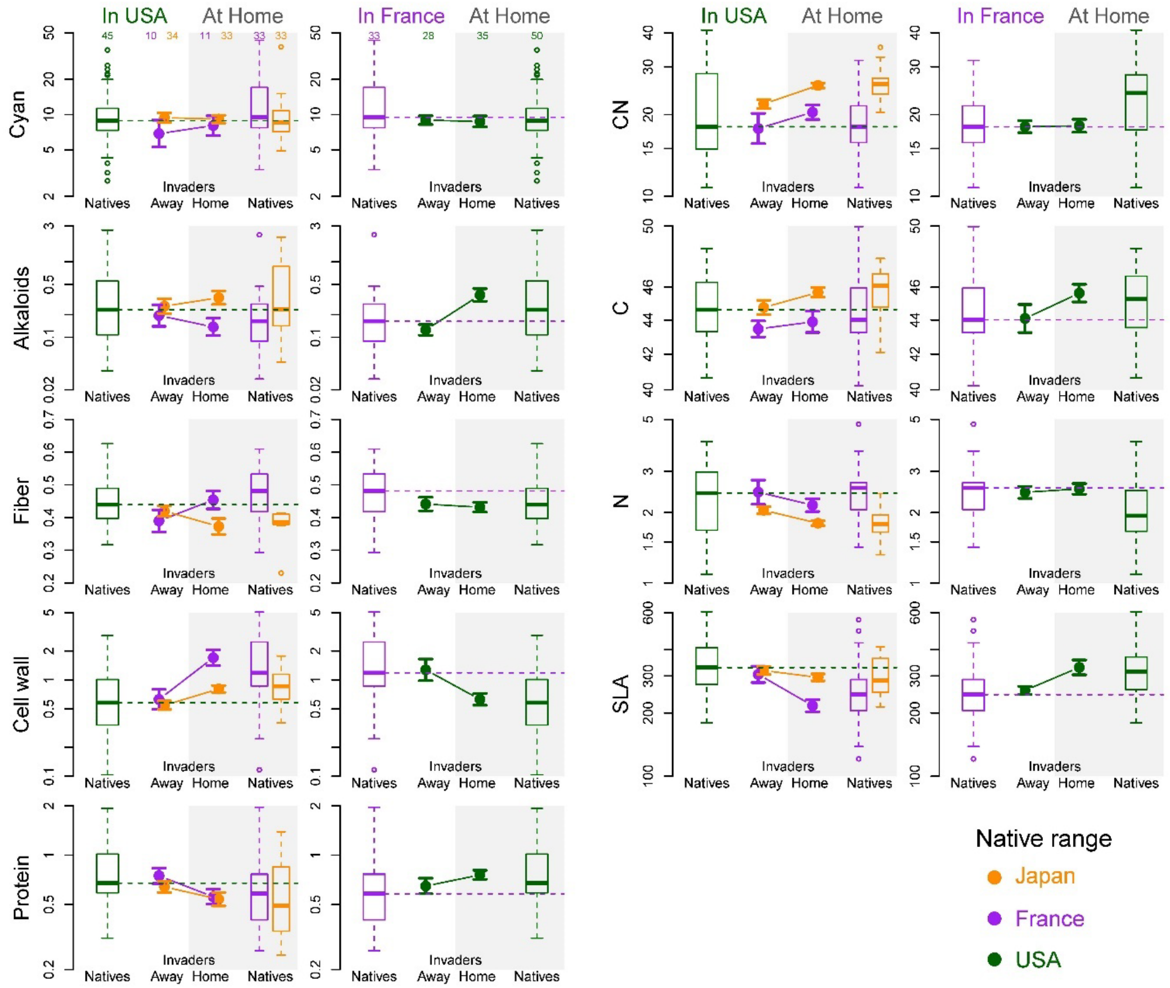

Figure S1. Shifts in leaf defensive traits across ranges in **woody** invasive species, compared to those of native co-occurring species in both the home and away ranges. Each panel shows a boxplot (quantiles and range) of trait values for native species of a given region, followed by the mean (+/- 1 s.e.) invader value present in the same region, separated by home region; the mean (+/- 1 s.e.) of those same invaders in their home range; and a boxplot of other (non-invasive) species in the home region. Samples sizes for each population are listed at the top of the first panel. Traits are listed in Table 2. Cyan values exclude *Prunus serotina* (see text).

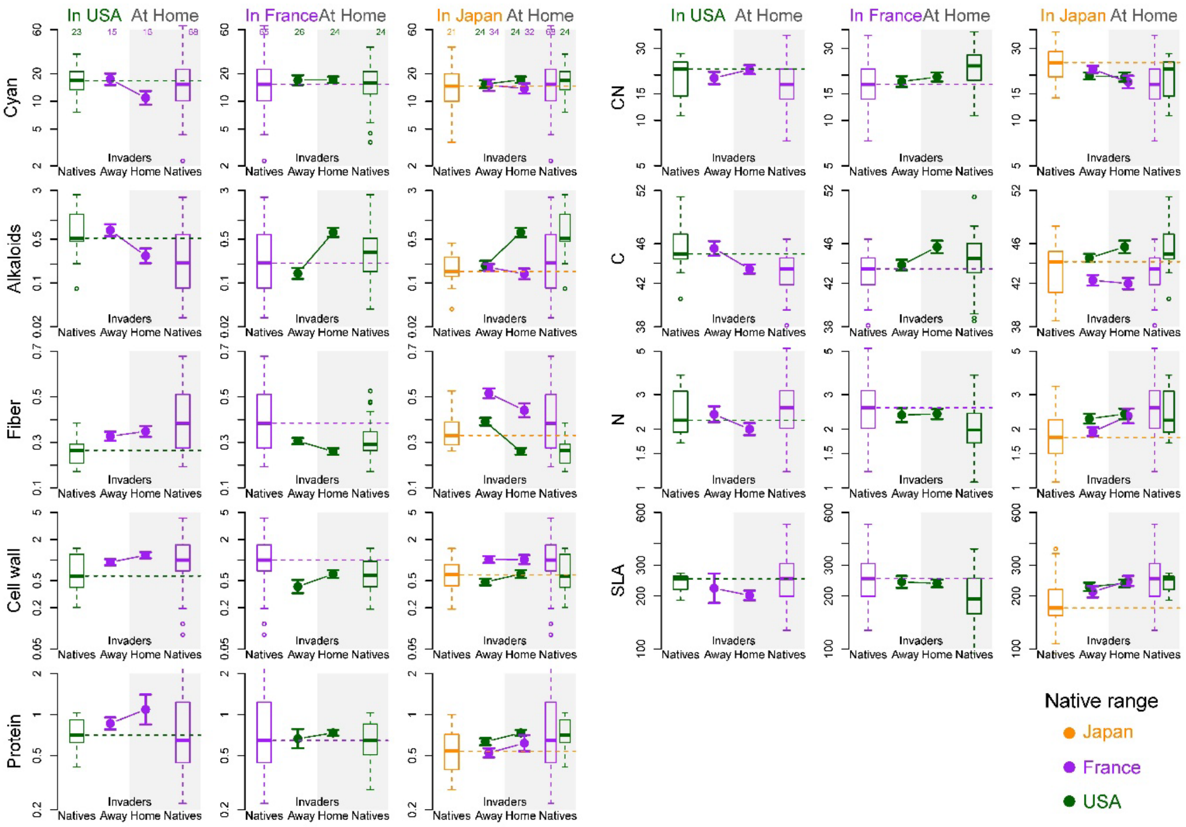

Figure S2. Shifts in leaf defensive traits across ranges in **herbaceous** invasive species, compared to those of native co-occurring species in both the home and away ranges. Each panel shows a boxplot (quantiles and range) of trait values for native species of a given region, followed by the mean (+/- 1 s.e.) invader value present in the same region, separated by home region; the mean (+/- 1 s.e.) of those same invaders in their home range; and a boxplot of other (non-invasive) species in the home region. Samples sizes for each population are listed at the top of the first panel. Traits are listed in Table 2.
